# Expression pattern of *SMAD8* in geese at different reproductive stages and its effect on reproductive physiology and egg production performance

**DOI:** 10.5455/javar.2026.m1022

**Published:** 2026-03-16

**Authors:** Daolun Yu, Ruina Zhang, Yanfei He, Kai Ge

**Affiliations:** 1 College of Life and Health, West Anhui University, Lu’an 237012, Anhui, China

**Keywords:** Wanxi White Goose (WWG), folliculogenesis, ovary and oviduct, breeder egg hatchability

## Abstract

**Objectives:** To elucidate the role of *SMAD8* in geese egg production, we analyzed the expression patterns of *SMAD8* in geese during the egg-laying and non-laying periods.

**Materials and Methods:** LDN-193189, a specific *SMAD8* inhibitor, was injected into the geese prior to the onset of egg-laying. Subsequently, the ovaries, oviducts, and serum of the geese at various reproductive stages were collected for subsequent detection and analysis.

**Results:** The results indicated that the development of ovaries and oviducts in geese during the egg-laying period was superior to that during the non-laying period. In addition, *SMAD8* expression in ovaries and oviducts of egg-laying geese was significantly higher than that in non-laying geese (*p* < 0.05). Injection of the *SMAD8* inhibitor LDN-193189 at a dose of 0.15 mg/kg body weight per day significantly inhibited *SMAD8* expression. Concomitantly, the expression levels of follicle-stimulating hormone receptor (*FSHR*) and luteinizing hormone receptor (*LHR*) expression in the ovaries and oviducts were significantly increased (*p* < 0.05). Furthermore, the average egg production of geese was significantly increased by 4.1 eggs (*p* < 0.05), while the hatchability of breeder eggs remained unchanged (*p* > 0.05). Collectively, this study demonstrates that *SMAD8* is differentially expressed at different reproductive stages in geese. Exogenous *SMAD8* inhibitors can regulate *in vivo SMAD8* expression, thereby modulating the expression of ovulation-related hormone receptors and ultimately improving the goose egg production.

**Conclusions:** These findings suggest that *SMAD8* may serve as a key intracellular regulator connecting reproductive organ development, hormone receptor abundance, and egg-laying performance in Wanxi White Goose (WWG) and indicate that targeted modulation of *SMAD8* activity could provide a novel strategy for boosting egg production in low-yield goose breeds without compromising hatchability.

## 1. Introduction

The goose is one of the traditional waterfowl species cultivated in China, and China ranks first globally in terms of goose meat consumption. Although the annual output of commercial geese in China has exceeded 500 million, it still fails to satisfy the market demand for goose products [1, 2]. Currently, the vast majority of goose breeds raised in China exhibit extremely low egg production. For instance, the WWG, a local breed in Anhui Province, East China, lays merely 20 to 25 eggs per year [3]. This extremely low egg production has become a prominent bottleneck restricting the sustainable development of the WWG industry. Therefore, improving goose egg production has emerged as the primary challenge in advancing China’s goose industry. In contrast to commercial chicken strains, which can produce over 300 eggs annually, the reproductive efficiency of most goose breeds remains considerably low. This significant gap underscores the urgency of clarifying the physiological mechanisms that limit goose egg production, thereby enabling the development of targeted strategies to enhance their reproductive performance.

The egg production of poultry is dependent on the development of female reproductive organs, among which the development of ovaries and oviducts is the most critical. The ovaries serve as the site where oocytes grow and mature, while the oviducts act as a conduit for oocyte transport and egg formation; both play an indispensable role in poultry reproduction. Studies have demonstrated that, compared with the non-laying period, the reproductive organs of female poultry undergo significant development during the egg-laying period to adapt to the physiological demands of egg production [4, 5, 6]. The hypothalamic-pituitary-gonadal (HPG) axis tightly regulates the growth and regression of ovaries and oviducts. Gonadotropins and ovarian steroid hormones act coordinately on these organs, thereby mediating follicular recruitment, maturation, and ovulation, as well as subsequent oviductal transport and eggshell formation.

*SMAD8* is an important transcription factor downstream of the BMP/SMAD signaling pathway [7]. In this pathway, BMP ligands bind to type I and type II serine/threonine kinase receptors and induce the phosphorylation of receptor-regulated SMADs (SMAD1/5/8). The phosphorylated SMADs then form complexes with SMAD4 and translocate into the nucleus, where they regulate the transcription of genes involved in cell proliferation, differentiation, and apoptosis. However, the precise regulatory mechanism remains unclear. Studies have indicated that *SMAD8* may participate in regulating animal ovarian development and ovulation by modulating the expression of factors associated with follicle maturation and ovulation, thereby influencing animal reproductive processes [8, 9]. Other studies have demonstrated that *SMAD8* is differentially expressed in follicles at distinct developmental stages during the egg-laying period, suggesting that *SMAD8* is involved in the establishment of the follicular hierarchy in poultry [10]. *SMAD8* also exhibits a certain dose-dependent manner in this regulatory process, yet its exact mechanism remains to be elucidated [11]. Furthermore, most current studies on *SMAD8* have been performed in mammals or *in vitro* cell systems, whereas information regarding its *in vivo* function in waterfowl, especially geese, remains limited. To date, no direct evidence has been reported regarding whether *SMAD8* expression directly affects the egg production performance of poultry. In addition, numerous studies have indicated that the morphologically adaptive development of animal reproductive organs during the reproductive period results from the coordinated actions of multiple internal factors. Among these, reproductive endocrine hormones and their receptors play indispensable roles, and their expression levels profoundly influence the reproductive processes of animals [12, 13, 14, 15].

This study focuses on the WWG, a breed characterized by extremely low egg production. Egged on by the observation of morphological differences in its reproductive organs between the egg-laying and non-laying periods, the expression levels of *SMAD8* in the ovaries and oviducts at different reproductive stages were quantitatively analyzed. LDN-193189, a specific inhibitor of bone morphogenetic protein (BMP) type I receptors, was injected into the wing vein of pre-laying geese, following which the expression level of *SMAD8* was dynamically monitored throughout the egg-laying period. In addition to tracking *SMAD8* expression levels, the experiment also detected the concentrations of follicle-stimulating hormone (FSH) and luteinizing hormone (LH) in the blood and the expression levels of *FSHR* and *LHR* in the ovaries and oviducts, as well as the egg production and hatchability of breeder eggs in geese during the post-injection egg-laying period. These monitored parameters were further compared with those of the control group (injected with normal saline), aiming to provide a scientific basis for improving goose egg production through the regulation of *SMAD8*. Collectively, these approaches were designed to clarify the role of *SMAD8* in the reproductive physiology of WWG and to verify whether pharmacological modulation of *SMAD8* activity can serve as a novel strategy to enhance egg production without impairing hatchability.

## 2. Materials and Methods

### 2.1. Ethical approval

This work was performed in the Laboratory of Poultry Reproductive Physiology, Department of Animal Science, West Anhui University, located in Lu’an, Anhui Province, China. All animal experiments conducted in this study were approved by the Institutional Animal Care and Use Ethics Committee of West Anhui University (License No. 202404002).

### 2.2. Animal grouping, tissue collection, serum sample collection

A total of 300 healthy 8-month-old breeding geese were selected for this study, all of which were provided by Anhui WWG Breeding Farm Co., Ltd. Among these geese, 240 female pre-laying geese and 60 male geese were grouped into 12 groups at a male-to-female ratio of 1:4, with 25 geese per group. Specifically, the geese were divided into three test groups (Groups A, B, and C) and one control group, with three replicates per group. Female pre-laying geese in test groups A, B, and C were continuously injected with the LDN-193189 inhibitor for 10 consecutive days at doses of 0.10 mg/kg body weight/day, 0.15 mg/kg body weight/day, and 0.20 mg/kg body weight/day, respectively. The injection volume of the inhibitor solution was consistent across all treated geese, at 300 μl per goose per day. The dosage of LDN-193189 was determined based on the inhibitor’s instructions and the average body weight (4 kg) of the geese during the experimental period. The feasibility of the inhibitor treatment protocol employed in this study was verified in a preliminary experiment. Geese in the control group were injected with the same volume of normal saline as the test groups every day for 10 consecutive days. All experimental geese were reared under natural light conditions, with free access to feed and drinking water throughout the experimental period.

At the peak of egg production in April of each year, five female geese were randomly selected from each group. Following brachial vein blood collection, sodium pentobarbital was slowly injected via the brachial veins at a dose of 30 mg/kg of body weight for euthanasia. Euthanasia was confirmed by the presence of respiratory arrest, cardiac arrest, and mydriasis within 1–2 min post-injection. The collected blood samples were centrifuged at 600 *×g* for 20 min at 4°C, and the upper serum was subsequently harvested and stored at –80°C for further analysis.

Immediately after euthanasia confirmation, the ovaries and oviducts within the abdominal cavity were promptly excised. The morphological characteristics of the reproductive organs during the breeding period were observed and photographed. Tissue samples (100 gm) were collected from the ovary and oviduct of each goose and frozen in liquid nitrogen for subsequent use, while the remaining tissues were fixed in paraformaldehyde solution. The same serum and tissue sampling procedures were repeated during the non-laying period of the geese. Additionally, the egg production of each goose was accurately recorded throughout the entire experimental period.

### 2.3. Tissue section, hematoxylin and eosin (H&E) staining

Ovary and oviduct samples fixed in paraformaldehyde for 48 h were embedded in paraffin wax, followed by sectioning into 5 μm-thick tissue slices using a manual rotary microtome (YDX RD-315, Liaoning, China). Briefly, the H&E staining procedure was performed as follows: paraffin sections were initially stained with hematoxylin for 3 min, subsequently differentiated in 1% hydrochloric acid-ethanol for several seconds, followed by rinsing with running tap water for 5 min, and then stained with eosin for 3 min. Finally, the sections were mounted with a neutral balsam, and tissue images were observed and captured using an Olympus BX61 microscope.

### 2.4. Immunohistochemical localization of SMAD8 expression in goose ovary and oviduct

Paraffin sections were deparaffinized and then placed in sodium citrate buffer (0.01 M, pH 6) for antigen retrieval, which was performed by microwave heating at 750 W for 1 min. After antigen retrieval, the sections were rinsed with phosphate-buffered saline (PBS) and subsequently blocked with Tris-HCl buffer (0.1 M, pH 7.5) containing 3% bovine serum albumin (BSA; Sangon, PR China) and 20% bovine serum (Sangon, PR China) for 30 min at room temperature. The blocked sections were incubated overnight with a primary antibody against SMAD8 (anti-SMAD8; Abcam) at 4°C. Following three washes with PBS, the sections were incubated with horseradish peroxidase (HRP)-conjugated secondary antibodies at room temperature for 1 h. Finally, the sections were subjected to color development according to the instructions of the DAB horseradish peroxidase color development kit (Beyotime, PR China), followed by hematoxylin counterstaining, dehydration, and mounting. All slides were observed under an Olympus BX61 fluorescence microscope, and the positive staining areas were captured and photographed.

### 2.5. RNA extraction, quantitative reverse-transcription polymerase chain reaction

Total RNA was extracted separately from goose ovarian and oviduct tissue samples following the standard protocol of the Trizol reagent method (Invitrogen, NM, USA). Subsequently, RNA quality was assessed using an ultramicro nucleic acid analyzer (Jiapeng 800, Shanghai, China). RNA samples with an absorbance ratio (*OD*_260/280_) ranging from 1.8 to 2.1 were considered qualified. Then, qualified RNA samples were immediately reverse transcribed into complementary DNA (cDNA) according to the instructions of the reverse transcription kit (Takara, Dalian, China) and stored at -80°C for subsequent use. Quantitative real-time PCR (qRT-PCR) was performed on a CFX Manager System (Bio-Rad, CA, USA) using the qRT-PCR kit (Takara, Dalian, China), with SYBR Green as the detection dye (Tiangen, Beijing, China). The qRT-PCR primers were designed based on the cDNA sequence of the target gene in goose, and glyceraldehyde-3-phosphate dehydrogenase (*GAPDH*) was used as the internal reference gene. Detailed primer sequence information is presented in Table 1. The qRT-PCR reaction system and operating procedures were performed as described previously [10]. Each sample was analyzed with at least five biological replicates, and the relative expression levels of target RNAs were calculated using the 2^–∆∆CT^) method [16].

**Table 1.** The primers for qRT-PCR are used in this study.

Gene	Forward (5’-3’)	Reverse (5’-3’)	Size (bp)
*SMAD1*	GAA CAC GAG GCG ACA CAT TG	ACT GTG GTC GGA TGG AAA CC	141
*SMAD5*	AGC CAG CTC TGG ACC ATC TA	AGG CAA CAG GCT GAA CAT CT	176
*SMAD8*	TG CAT TAA CCC CTA CCA TTA CCG GC	GGA TGC TGG AAA GAA TCG GGG TAG	129
*ERα*	TGG AGC CAG TGC TAC CTG TGA C	GCA GCC TTC GCA TGA CCA GAC	173
*FSHR*	CCA ATG CCA CTG AAC TGA GA	CCT CCA AGG CAT CAT TCT GT	117
*LHR*	AGA CGC GTT TAA TCC CTG TG	GCT GGT TAT GAG GAC GAG GA	121
*PR-A*	ATC ATC GTT CTA TTC ACT GT	CTC GTT CTC ATC TCA TCA A	168
*GAPDH*	TGG TGC TAA GCG TGT CAT CAT CTC	ACT GTC TTC TGT GTG GCT GTG ATG G	222

### 2.6. Protein extraction, western blotting (WB)

Total protein was extracted separately from goose ovarian and oviduct tissue samples using RIPA lysis buffer (Shenggong Biotechnology, Shanghai, China), and the concentration of protein samples was quantified with a BCA kit (Boster, Wuhan, China). The antibody information used in WB was as follows: mouse anti-SMAD1/5/8 (Abcam, MA, USA) and mouse anti-GAPDH (Santa Cruz Biotechnology, CA, USA), and goat anti-mouse HRP-conjugated secondary antibody (Cell Signaling Technology, MA, USA). WB experiments were performed using a WB kit (Shenggong Biotechnology, Shanghai, China) in accordance with the manufacturer’s instructions. The experimental procedure was briefly described as follows: WB was conducted via the wet transfer method using a Mini Trans-Blot system (Tianneng, Shanghai, China). A total of 20 μg of denatured protein samples was mixed with loading buffer and loaded onto the sample wells of a 10% SDS-PAGE gel. After electrophoresis, the separated proteins on the gel were transferred to a polyvinylidene difluoride (PVDF) membrane, which was then blocked for 1 h with 5% skimmed milk in Tris-buffered saline (TBS) containing 0.1% Tween-20 at room temperature. Subsequently, the PVDF membrane was incubated with the primary antibody overnight at 4°C, followed by incubation with the secondary antibody for 1 h at room temperature. The blot bands were detected using an ECL detection system according to the WB kit protocol. Each protein experiment was repeated five times. The gray intensities of the bands were measured using ImageJ software (National Institutes of Health, MD, USA) and normalized to GAPDH levels.

### 2.7. Serum reproductive hormone concentration detection

The concentrations of estradiol (E_2_), progesterone (P_4_), follicle-stimulating hormone (FSH), and luteinizing hormone (LH) in serum were determined by enzyme-linked immunosorbent assay (ELISA), and the experiments were performed in accordance with the manufacturer’s instructions of the goose-derived ELISA kits (Halin Biotechnology, Shanghai, China).

### 2.8. Egg production and hatchability of breeder eggs

The average egg production per goose during the experimental period was calculated based on the total egg production and the average daily number of female geese reared. Subsequently, all collected eggs were incubated in a programmable automatic incubator (Yiai 19200, Anhui, China). At the end of incubation, the total number of goslings was counted, and the hatchability of breeder eggs was calculated as the ratio of the total number of goslings to the total number of fertilized eggs incubated.

### 2.9. Statistical analysis

The results were presented as the mean ± standard deviation (SD) of five repetitions. Experimental data from five birds were analyzed using SPSS 22.0 software (SPSS Inc., Shanghai, China). Prior to statistical analysis, normality of data was verified using the Shapiro-Wilk test, and homogeneity of variance was assessed via the Brown-Forsythe test. Statistically significant differences among groups were determined using Student’s *t*-test or one-way analysis of variance (ANOVA) followed by Duncan’s multiple comparison test. Investigators were blinded to the experimental conditions during both data collection and analysis. Statistical significance was accepted at *p* < 0.05, and significant differences are indicated by different lowercase superscript letters in figures and tables.

## 3. Results

### 3.1. Morphology of goose ovary and oviduct at different reproductive stages

**Figure 1.**
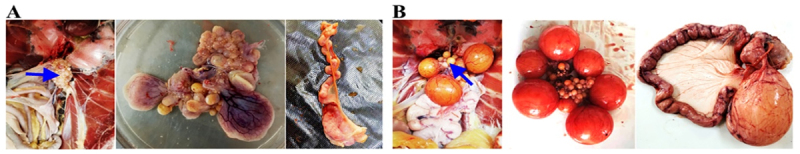
Morphology of the ovary and oviduct in geese during different reproductive periods. (A) Non-laying period. (B) Egg-laying period. In both images, the left (blue arrow), middle, and right structures indicate the intra-abdominal ovary in situ, the isolated ovary, and the oviduct, respectively.

**Figure 2.**
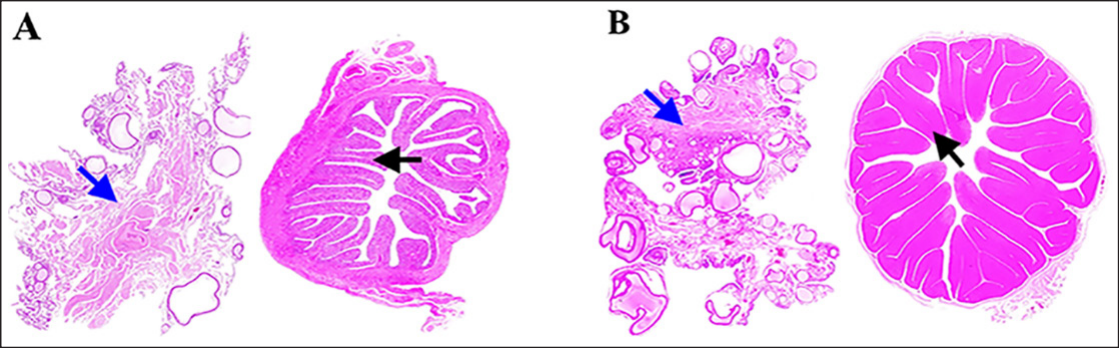
Histological structure of the ovary and oviduct in geese during different reproductive periods. (A) Non-laying period. (B) Egg-laying period. In both images: left, ovary; right, oviduct. Blue arrow, ovarian parenchyma; black arrow, oviductal mucosal fold. Scale bar: 200 μm.

Ovaries and oviducts were collected from WWG during the egg-laying and non-laying periods, respectively, to elucidate morphological differences between the two reproductive stages. The results showed that the ovaries and oviducts of geese during the non-laying period were smaller in volume and paler in color (Figure 1A). In contrast, marked morphological changes were observed during the egg-laying period: the reproductive organs exhibited a sharp increase in volume, and pre-hierarchical and pre-ovulatory follicles of various sizes and quantities were distributed on the ovarian surface. The ovarian weight during the egg-laying period increased to more than twice that during the non-laying period. Abundant hyperemic blood vessels were clearly visible on the oviductal wall, giving the oviduct a pinkish appearance, and both oviductal length and wall thickness were significantly greater than those in the non-laying period (Figure 1B). Further H&E staining revealed that during the non-laying period, ovarian parenchyma was poorly developed and relatively loose, with weak connections between adjacent tissues and distinct gaps under light microscopy. Meanwhile, the oviductal diameter was small, and the mucosal folds in the oviductal lumen were thin and poorly developed, resulting in large spaces between the folds (Figure 2A). During the egg-laying period, the ovarian parenchyma became dense, with numerous round or subrounded follicles at different developmental stages. The oviductal lumen diameter increased to more than twice that of the non-laying period. The mucosal folds were well-developed, tall, and wide, with almost no gaps between adjacent folds (Figure 2B). Comparative analyses of the developmental changes in ovaries and oviducts between the non-laying and laying periods are summarized in Table 2.

**Table 2.** Comparison of developmental changes in ovaries and oviducts of geese during non-laying and egg-laying periods.

Period	Oviduct					Ovary	
	Color	Shape	Vessel	Average lumen diameter (μm)	Mucosal fold height (μm)	Average weight (gm)	Number of follicles
Egg-laying	Pink	Thick and long	Obviously visible	9100	1230–4240	90	More
Non-laying	Pale	Thin and short	Indistinguishable	4300	650–1370	43	Fewer

These results indicated that the morphology of the ovaries and oviducts of geese during the egg-laying period exhibited obvious developmental advantages compared with those during the non-laying period, and such changes provide sufficient morphological preparation for egg production. Although this section was mainly descriptive, highly consistent morphological characteristics were observed in all individuals within each group (*n* = 5), confirming the reliability of the observed differences between egg-laying and non-laying geese. These structural changes are consistent with the typical reproductive tract activation reported in other poultry species at the onset of laying and are driven by the coordinated actions of multiple endocrine hormones *in vivo*. Therefore, these morphological alterations provide a solid structural basis for interpreting the molecular and endocrine data obtained in subsequent experiments.

### 3.2. Expression of SMAD8 in the ovary and oviduct of geese

**Figure 3.**
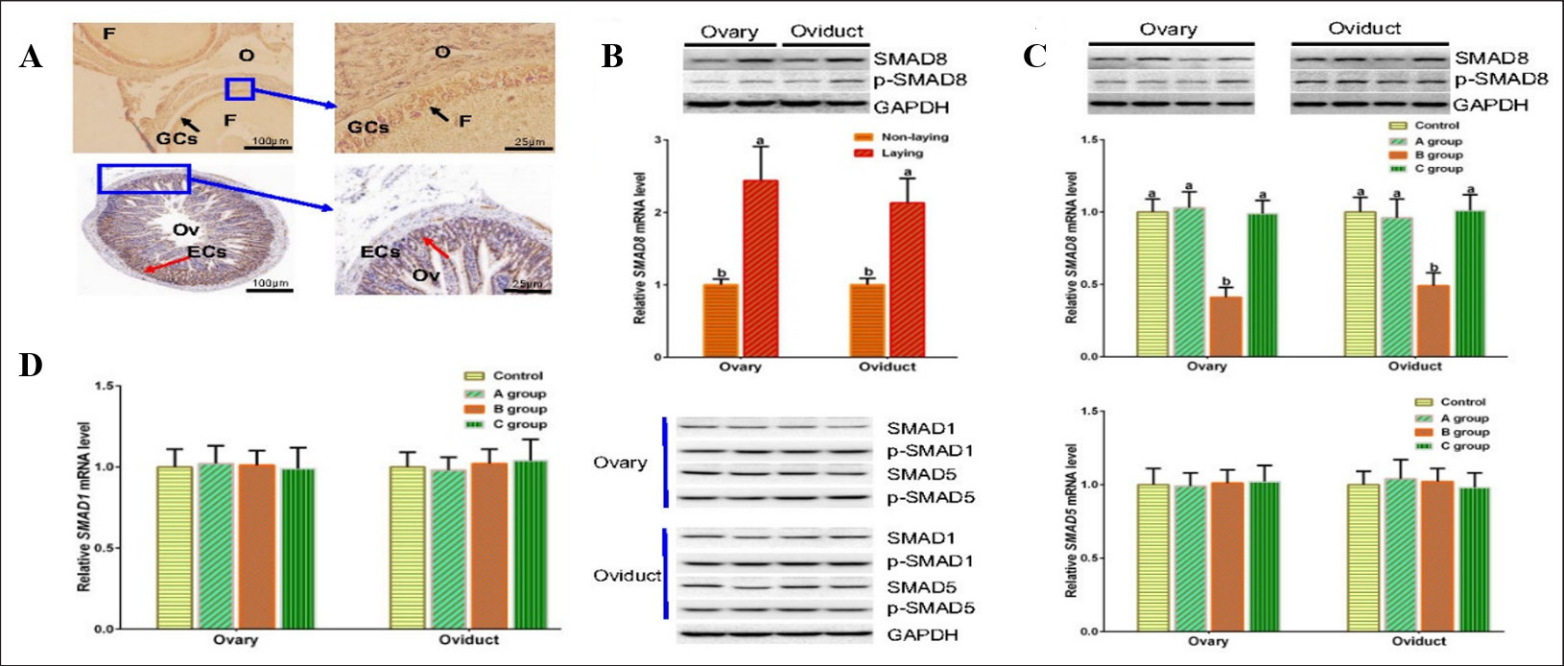
Localization and expression of SMAD8 in goose ovary and oviduct (*n* = 5). (A) Immunohistochemical localization of SMAD8 in follicular granulosa cells and oviductal epithelial cells. F, follicle; O, ovary; GCs, granulosa cells; ECs, epithelial cells; Ov, oviduct. Black arrow, positive signal in granulosa cells; red arrow, positive signal in oviductal epithelial cells. (B) SMAD8 expression levels during non-laying and laying periods. (C) SMAD8 expression levels after inhibitor injection. (D) SMAD1/5 expression levels after inhibitor injection. Different lowercase letters indicate significant difference (*p* < 0.05). *n* is the number of animals.

To investigate the differential expression of *SMAD8* at different reproductive stages in geese, immunohistochemistry was performed to localize SMAD8 protein in follicles and oviducts, and qRT-PCR and WB were used to detect and analyze SMAD8 expression in ovaries and oviducts during the non-laying and egg-laying periods. Immunohistochemical results revealed that SMAD8 protein was expressed in follicular granulosa cells and oviductal epithelial cells (Figure 3A). qRT-PCR and WB analyses demonstrated that SMAD8 expression levels in the ovaries and oviducts of low-yield WWG were significantly higher during the egg-laying period than during the non-laying period (*p* < 0.05, Figure 3B). To determine whether exogenous gene inhibitors affect *SMAD8* expression *in vivo, SMAD8* expression levels were compared between the *SMAD8* inhibitor LDN-193189 injection group and the control group. Administration of the *SMAD8* inhibitor at 0.15 mg/kg body weight per day significantly inhibited *SMAD8* expression in goose ovaries and oviducts *in vivo* (*p* < 0.05, Figure 3C). These results indicated that *SMAD8* was differentially expressed between the non-laying and egg-laying periods, and that an exogenous inhibitor could regulate *SMAD8* expression *in vivo*. To exclude potential off-target effects of LDN-193189 on other SMAD proteins in the BMP/SMAD signaling pathway, the expression of SMAD1/5 was also examined. Results showed that SMAD1/5 expression levels were not affected by LDN-193189 treatment (Figure 3D). Notably, the expression patterns of SMAD8 were consistent at both mRNA and protein levels, as well as between ovarian and oviductal tissues, supporting the reliability of the present findings. To further clarify the mechanism by which the BMP type I receptor inhibitor LDN-193189 modulates signal transduction, the levels of p-SMAD1/5 in the ovary and oviduct were also detected. The expression pattern of p-SMAD1/5/8 was consistent with that of total SMAD1/5/8, suggesting that LDN-193189 exerts its biological function mainly by targeting the expression of *SMAD8* (Figures 3B, C, D). The marked downregulation of *SMAD8* achieved with the intermediate dose of LDN-193189 indicates that this inhibitor can effectively reach target tissues and regulate the BMP/SMAD pathway in geese *in vivo*.

### 3.3. Reproductive hormones in goose blood across reproductive stages

E_2_, P_4_, FSH, and LH are representative reproductive hormones involved in regulating follicular development and ovulation in poultry. To determine the differences in the levels of these hormones at different reproductive stages in geese, ELISA was performed on serum samples collected from geese during the non-laying and egg-laying periods. As shown in Table 3, the levels of these reproductive hormones in geese were generally significantly higher during the laying period than during the non-laying period (*p* < 0.05). In addition, serum hormone levels were also measured following the injection of LDN-193189. As shown in Table 4, E_2_ levels increased only in groups B and C, but these increases were not statistically significant compared with the control group; no significant changes in P_4_, FSH, or LH levels were observed after inhibitor administration. These results indicated that the levels of reproductive hormones in goose blood were significantly higher during the egg-laying period than during the non-laying period. These hormones not only play a crucial role in regulating the initiation, maturation, and ovulation of follicles in goose ovaries but are also directly associated with the progressive development of goose reproductive organs during the egg-laying period.

**Table 3.** Hormone levels in the blood of geese at different stages of reproduction.

Period	E_2_ (pmol/l)^1^	FSH (U/l)	LH (pg/ml)	P_4_ (pmol/l)
Egg-laying	697.83 ± 83.47^a, 2^	4.04 ± 0.51^a^	164.51 ± 17.14^a^	47.34 ± 5.89^a^
Non-laying	418.64 ± 61.83^b^	2.71 ± 0.22^b^	43.47 ± 4.58^b^	10.26 ± 1.75^b^

^1^ E_2_, estradiol; FSH, follicle-stimulating hormone; LH, luteinizing hormone; P_4_, progesterone. The values are presented as the mean ± SD. ^2^ Different letters in the same column of shoulder markers represent significant difference (*p* < 0.05).

**Table 4.** Hormone levels in the blood of geese after injection of inhibitor.

Group	E_2_ (pmol/l)^1^	FSH (U/l)	LH (pg/ml)	P_4_ (pmol/l)
Group A	699.37 ± 81.07	3.96 ± 0.77	162.47 ± 21.23	49.02 ± 6.81
Group B	757.34 ± 90.16	4.05 ± 0.95	161.08 ± 17.56	51.30 ± 7.25
Group C	729.14 ± 92.21	4.13 ± 0.73	155.79 ±18.91	51.05 ± 6.78
Control group	711.55 ± 88.13	4.19 ± 0.81	162.19 ± 20.78	50.33 ± 6.55

The blood samples of WWG were collected during the egg-laying period. The absence of shoulder letters in the same column indicates no significant difference (*p* > 0.05). ^1^E_2_, estradiol; FSH, follicle-stimulating hormone; LH, luteinizing hormone; P_4_, progesterone. The values are presented as the mean ± SD.

### 3.4. Reproductive hormone receptor expression in goose ovaries and oviducts

**Figure 4.**
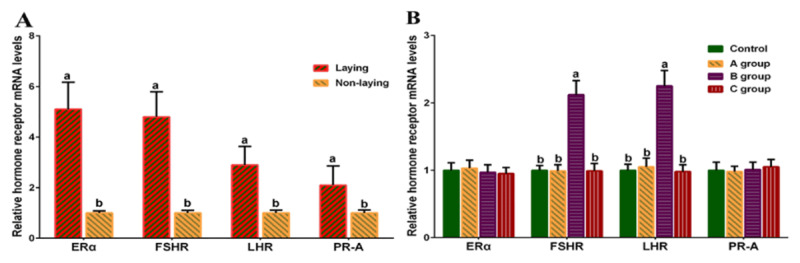
Expression of reproductive hormone receptors in the goose ovary and oviduct at different reproductive periods (*n* = 5). (A) Reproductive hormone receptor expression levels during non-laying and laying periods. (B) Reproductive hormone receptor expression levels after inhibitor injection. Different lowercase letters indicate significant differences (*p* < 0.05).

It is well known that the regulatory effects of reproductive hormones on avian ovulation are mediated by their corresponding receptors. Accordingly, we quantitatively analyzed mRNA expression of *ERα, PR-A, FSHR*, and *LHR* in the ovaries and oviducts of geese across different reproductive stages. As shown in Figure 4A, the expression levels of these genes were significantly higher during the egg-laying period than in the non-laying period (*p* < 0.05).

Following *in vivo* injection of LDN-193189 to inhibit *SMAD8* expression, significantly increased expression of *FSHR* and *LHR* in the ovaries and oviducts during the laying period was observed only in Group B (*p* < 0.05), whereas no significant differences were detected in the other treatment groups or for other hormone receptors (*p* > 0.05, Figure 4B). These results demonstrated that reproductive hormone receptors were highly expressed during the egg-laying period in geese, implying their close involvement in the regulation of goose egg production.

Furthermore, suppression of SMAD8 expression using the inhibitor significantly elevated the expression of hormone receptors associated with follicular development and ovulation, suggesting that the *SMAD8* inhibitor may exert a potential promoting effect on goose ovulation. The synchronized upregulation of *FSHR* and *LHR* in both ovaries and oviducts of Group B is consistent with an overall enhancement of tissue sensitivity to gonadotropins. Combined with the observation that circulating hormone concentrations remain unchanged, these findings support the notion that SMAD8 functions as an intracellular modulator of gonadotropin signaling and that partial inhibition can selectively upregulate key receptors through specific mechanisms without disrupting overall endocrine balance.

### 3.5. Effects of the SMAD8 inhibitor on egg production in geese

**Figure 5.**
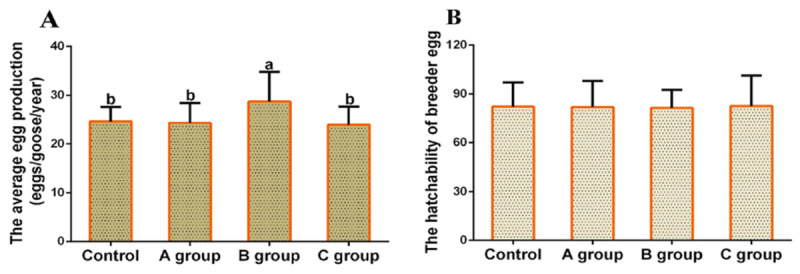
Effects of *SMAD8* inhibitor on goose egg production and breeder egg hatchability. (A) Average egg production (*n* = 5). (B) Hatchability of breeder eggs. Different lowercase letters indicate a significant difference (*p* < 0.05). No lowercase letters indicate no significant difference (*p* > 0.05).

To further investigate the effect of the *SMAD8* inhibitor on goose egg production, the average egg production and breeder egg hatchability of geese were recorded throughout the experimental period. As shown in Figure 5A, the average egg production was 24.3 in Group A, 28.7 in Group B, 23.9 in Group C, and 24.6 in the control group. Notably, the average egg production of Group B was significantly higher than that of the other experimental groups and the control group (*p* < 0.05). According to Figure 5B, the breeder egg hatchability was 81.9% in Group A, 82.1% in Group B, 81.5% in Group C, and 82.4% in the control group, with no significant differences observed among the four groups (*p* > 0.05). These results indicate that the *SMAD8* inhibitor injected at a dose of 0.15 mg/kg body weight per day (Group B) can significantly increase goose egg production, while the administration of the *SMAD8* inhibitor had no adverse effects on the breeder egg hatchability of geese. From the perspective of practical breeding, an increase of approximately 4 eggs per bird in low-egg-production breeds such as the WWG constitutes a meaningful improvement in annual productive performance. The consistency between the higher egg production in Group B and the concurrent upregulation of *FSHR* and *LHR* expression in their reproductive organs further strengthens the conclusion that moderate inhibition of *SMAD8* activity can enhance egg-laying performance while maintaining normal breeder egg hatchability.

## 4. Discussion

The reproductive activity of birds is affected by various factors, and their reproductive performance is not only related to genetic factors but also closely associated with environmental, physiological, and other factors. The reproductive physiological development of birds includes the maturation of reproductive organs and germ cells, which is coordinately regulated by multiple endocrine hormones *in vivo* [17, 18, 19]. Studies have demonstrated that the developmental status of avian reproductive organs differs significantly across reproductive stages [20, 21].

These developmental changes establish a favorable internal environment for the full expression of reproductive potential and represent a prerequisite for normal and timely egg-laying in birds [22]. Egg formation in birds relies on a fully developed reproductive tract as the physiological platform. The developmental state of the reproductive tract profoundly influences avian reproductive activity and is closely correlated with egg quantity and quality [23, 24]. Therefore, exploring the developmental characteristics of the reproductive tract at different reproductive stages in poultry is of great significance. In this study, we compared the developmental changes in ovaries and oviducts of geese at different reproductive stages and found that the development of reproductive organs was markedly superior during the egg-laying period than in the non-laying period. Morphologically, these changes included a significant increase in ovarian volume, numerous follicles on the ovarian surface, elongation and thickening of the oviduct, expansion of the oviductal lumen, and thickening of the oviductal wall.

Histologically, the ovarian parenchyma changed from loose to compact, the number of follicles embedded in the parenchyma increased substantially, and the mucosal folds within the oviduct developed from rudimentary to well-developed. These observations in geese are generally consistent with previous reports in other poultry species [25, 26]. In WWG, a breed characterized by inherently low egg production, such pronounced structural remodeling of the reproductive tract is particularly important, because even modest improvements in follicular recruitment and oviduct function may lead to measurable improvements in egg-laying performance. The present findings thus provide morphological evidence that low-yield native breeds retain sufficient reproductive organ plasticity to respond to endocrine and molecular regulatory signals during the egg-laying period. When seasonally breeding animals enter the breeding season, their reproductive organs undergo a natural cyclic development that initiates the egg-laying cycle. The morphological maturation of reproductive organs prepares the internal environment for egg formation in poultry and fetal development in oviparous species.

The BMP/SMAD signaling pathway is critical for the regulation of animal reproduction. During follicular development, this pathway crosstalks with the NOTCH and PI3K/Akt pathways. In terms of steroidogenesis, it exerts an inhibitory effect in mammals but a stimulatory effect in avian species. In addition, BMP/SMAD also interacts with the Wnt/β-catenin, AMPK, JAK/STAT, and Hippo/YAP pathways, forming a sophisticated regulatory network that collectively modulates follicular development, steroid synthesis, and ovarian homeostasis [27, 28]. SMAD family members regulate fertility distinctly: *SMAD1*-deficient mice die embryonically [29]; *SMAD2* knockdown reduces P450 aromatase [30]; *SMAD3*-deficient mice are infertile with anovulation [31, 32]; and *SMAD4* silencing inhibits porcine granulosa cell proliferation [33]. *SMAD8* regulates cell development and follicular ovulation dose-dependently, but its mechanism remains unclear [11, 34]. To clarify the potential role of the *SMAD8* in avian reproduction, we quantified its expression in goose ovaries and oviducts during non-laying and egg-laying periods. We found that *SMAD8* expression was significantly higher in the egg-laying period, indicating its involvement in reproductive organ development. This pattern suggests that *SMAD8* is not a simple on/off switch but rather participates in a finely tuned regulatory network in which both insufficient and excessive signaling may be detrimental to optimal follicular development. It is therefore reasonable to speculate that there exists an appropriate physiological “window” of *SMAD8* activity during the egg-laying period, within which granulosa cell proliferation and follicular selection are most efficient. Inspired by the dose-dependent effects found in previous studies, we tried to regulate *SMAD8* expression in geese during the egg-laying period by using the exogenous *SMAD8* inhibitor LDN-193189. The results showed that using the *SMAD8* inhibitor LDN-193189 in egg-laying geese, appropriate doses significantly downregulated *SMAD8* and increased egg production by 4.1 eggs / bird / year. The apparent paradox—higher *SMAD8* expression during egg-laying yet improved productivity with partial pharmacological inhibition—may reflect differences between physiological and experimental pathway regulation, where moderate suppression of overactivated signals restores folliculogenesis-favorable activity. Consistent with previous cell-based studies demonstrating BMP/SMAD signaling modulates granulosa cell proliferation and ovulatory gene expression, our findings show systemic *SMAD8* inhibition enhances whole-bird egg production [9, 11]. Further hatching experiments showed inhibitor injections did not affect goose hatchability, indicating *SMAD8* inhibitors have production potential for improving egg production and providing a new approach to enhance goose reproductive capacity. However, LDN-193189 (a type I BMP receptor inhibitor) lacks complete *SMAD8* specificity, so off-target effects on other SMADs or BMP-responsive pathways cannot be excluded. Future investigations employing more selective molecular approaches, such as targeted gene editing or tissue-specific *SMAD8* knockdown, will be essential to elucidate the precise contribution of this transcription factor to goose reproductive physiology. From an avian reproductive physiology perspective, modulating follicle recruitment via molecular inhibition is a double-edged sword, potentially triggering premature ovarian failure and reproductive dysfunction. Notably, this study only covered one goose breeding season, leaving unevaluated its effects on subsequent production sustainability. Whether observed improvements reflect genuine enhanced reproductive efficiency or transient ovarian reserve overdraw remains unclear; thus, long-term observations across multiple breeding seasons are warranted to confirm the regulatory strategy’s physiological safety and stability.

**Figure 6.**
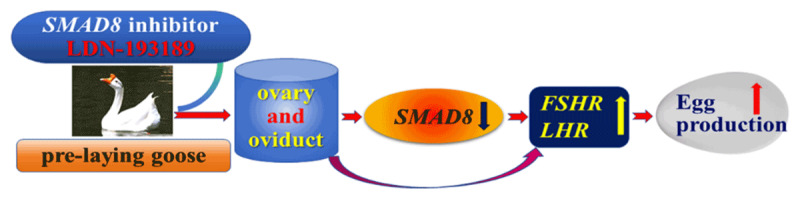
A model for *SMAD8* inhibitors regulating goose egg production. Inhibiting *SMAD8* during the laying period upregulates *FSHR* and *LHR* expression, thereby enhancing egg production. Upward arrows indicate a significant increase; downward arrows, a significant decrease.

Numerous studies confirm that avian reproductive activities depend on the combined action of multiple endocrine hormones [15, 35]. Pituitary hormones FSH, LH, and ovarian steroid hormones E2 and P_4_ play key roles throughout the process [9, 35], and their synergy promotes ovarian/follicular development and E2 and P_4_ expression [36, 37, 38], which further interact with other factors to facilitate follicular maturation and oocyte expulsion [39, 40]. In this experiment, blood reproductive hormone levels in geese were significantly higher during the egg-laying period than the non-laying period (E_2_, P_4_, FSH, LH), consistent with Zhao et al.’s findings on Sichuan White Geese [41]. This confirms that WWG follows a conserved reproductive endocrine axis similar to other goose breeds and poultry. Concurrent detection showed significantly higher expression of *ERα, PR-A, FSHR*, and *LHR* in goose ovaries and oviducts during the egg-laying period, with consistent trends in both organs. This indicates that high reproductive hormone levels stimulate corresponding receptor expression, which in turn promotes reproductive organ development via receptor mediation, forming a positive feed-forward loop to sustain follicular recruitment and ovulation. Notably, injection of the *SMAD8* inhibitor did not alter circulating reproductive hormone levels but markedly upregulated *FSHR* and *LHR*—pivotal hormone receptors governing follicle maturation and ovulation [42, 43, 44]—which ultimately contributed to enhanced egg production in geese. However, this benefit may not be solely due to direct *SMAD8* inhibition but also indirect regulatory pathways, requiring further functional validation to confirm the causal mechanism. The absence of systemic hormonal changes but local receptor alterations suggests *SMAD8* inhibition modulates ovarian and oviductal responsiveness rather than systemic endocrine output, highlighting intra-ovarian signaling importance and the potential of *SMAD8* targeting to improve reproductive efficiency without disrupting hormonal homeostasis. The experimental model is shown in Figure 6.

## 5. Conclusions

We compared ovarian and oviductal developmental changes and *SMAD8* differential expression in geese at different reproductive stages. Pharmacological inhibition of *SMAD8* using the BMP type I receptor inhibitor LDN-193189 in pre-laying geese significantly reduced *SMAD8* expression in ovaries and oviducts during egg-laying and markedly increased egg production without impairing hatchability. Quantitative detection of blood reproductive hormone concentrations and ovarian and oviductal *ERα, PR-A, FSHR, and LHR* expression showed that improved egg-laying in the inhibitor group was associated with upregulated *FSHR* and *LHR* in reproductive tissues, not with significant changes in circulating E_2_, P_4_, FSH, or LH—suggesting *SMAD8* may regulate reproductive tract sensitivity to gonadotropins. This study explains the correlation between ovarian and oviductal morphological changes, functional adaptation, and hormone regulation, providing a scientific basis for further research on the physiological regulatory mechanisms of goose egg production.

## Data Availability

The data presented in this study are available from the corresponding author upon reasonable request.

## List of abbreviations

WWG, Wanxi White Goose; HPG, hypothalamic-pituitary-gonadal; H&E, hematoxylin and eosin; PBS, phosphate-buffered saline; BSA, bovine serum albumin; HRP, horseradish peroxidase; cDNA, complementary DNA; E_2_, estradiol; P_4_, progesterone; FSH, follicle-stimulating hormone; LH, luteinizing hormone; *FSHR*, follicle-stimulating hormone receptor; *LHR*, luteinizing hormone receptor; *ERα*, estrogen receptor alpha; *PR-A*, progesterone receptor type A; *GAPDH*, glyceraldehyde-3-phosphate dehydrogenase; qRT-PCR, quantitative reverse-transcription polymerase chain reaction; ELISA, enzyme-linked immunosorbent assay; WB, western blotting; PVDF, polyvinylidene difluoride; SD, standard deviation; ANOVA, one-way analysis of variance.
